# Management of Poor-Grade Aneurysmal Subarachnoid Hemorrhage and Key Pearls for Achieving Favorable Outcomes: An Illustrative Case

**DOI:** 10.7759/cureus.33217

**Published:** 2023-01-01

**Authors:** Michael A Bamimore, Seung J Lee, Carlos Perez Vega, Nolan Brown, Julian L Gendreau, Rana Hanna Al Shaikh, Suren Jeevaratnam, William D Freeman

**Affiliations:** 1 Department of Neurological Surgery, Mayo Clinic, Jacksonville, USA; 2 Department of Neurological Surgery, University of California Irvine, Orange, USA; 3 Department of Biomedical Engineering, Johns Hopkins Whiting School of Engineering, Baltimore, USA; 4 Department of Neurologic Surgery, Mayo Clinic, Jacksonville, USA

**Keywords:** poor-grade, hydrocephalus, intracranial hypertension, sub-arachnoid hemorrhage, aneurysm

## Abstract

Poor-grade aneurysmal subarachnoid hemorrhage (aSAH) is associated with high patient mortality. Despite recent advances in management strategies, the prognosis for poor-grade aSAH remains dismal. We present a challenging case of a patient presenting with poor-grade aSAH. A 46-year-old female presented to the emergency department after losing consciousness following a sudden headache. The examination showed a dilated left pupil and a Glasgow Coma Scale of 4. Imaging revealed a ruptured anterior communicating artery (ACoM) aneurysm, after which the patient was subsequently taken to the neuro-interventional radiology suite. We showed that carefully managing blood pressure and intracranial pressure (ICP) makes it possible to achieve a favorable outcome and reduce the risk of secondary brain injury in aSAH, regardless of patient presentation. We propose maintaining blood pressure at <160 mmHg prior to intervention, after which it can be permitted to increase to 160-240 mmHg for the purpose of preventing vasospasm. Additionally, transcranial doppler (TCD) is essential to detect vasospasm due to the subtility of symptoms in patients with aSAH. Once identified, vasospasm can be successfully treated with balloon angioplasty. Finally, targeted temperature management (TTM), mannitol, hypertonic saline, and neuromuscular paralysis are essential for the postoperative management of ICP levels.

## Introduction

Aneurysmal subarachnoid hemorrhage (aSAH) occurs from a ruptured intracranial aneurysm with an estimated prevalence of about 70,000 cases per year in the United States [1,2]. Risk factors for aSAH include hypertension, smoking, and a previous family history of aSAH [1,2]. Symptoms of an aneurysm rupture include a sudden, excruciating headache ("thunderclap"), loss of consciousness from a sudden spike in intracranial pressure (ICP), seizure, nausea, vomiting, and a decreased level of consciousness (LOC). The prognosis of aSAH was historically graded on the Hunt Hess scale and recently the World Federation of Neurological Surgeons (WFNS) grading scale has been utilized. Those patients who are awake with a headache and a relatively preserved LOC on the Glasgow Coma Scale (GCS) scores such as >12, are typically designated "good-grade" [1-3]. The prognosis for aSAH is correlated with the initial WFNS score, with rates of survival estimated to be as high as 65% [1-4].

In the present study, the authors present a challenging case of a patient who presented with a poor-grade aSAH complicated by vasospasm and an elevated ICP and who ultimately made a remarkable recovery. We also review the most current literature regarding management strategies to decrease the risks of the four most common causes of secondary brain injury: (1) aneurysm re-rupture, (2) hydrocephalus, (3) cerebral salt wasting, and (4) delayed cerebral ischemia/vasospasm.

## Case presentation

**Figure 1:**
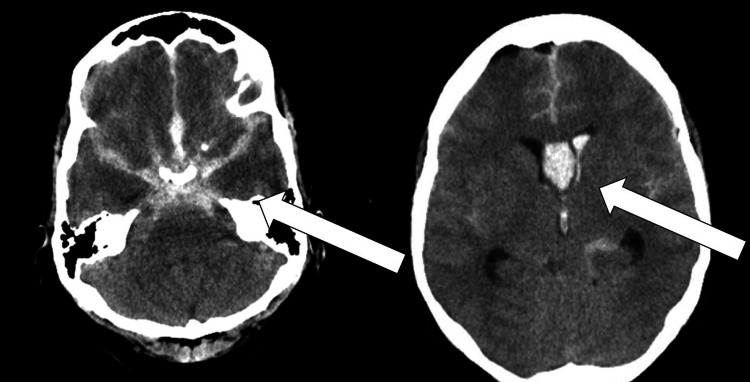
Initial CT imaging of the identified diffuse subarachnoid hemorrhage (left) and intraventricular extension (right). CT: computed tomography.

A 46-year-old female was brought to a local emergency department after a loss of consciousness following a sudden headache. On examination, she had a GCS of 4 (extensor posturing), a left pupil larger than the right, and a positive urine study for cocaine. Computed tomography (CT) revealed a diffuse subarachnoid hemorrhage with intraventricular hemorrhage (Figure 1). CT angiography revealed an anterior communicating artery (ACoM) aneurysm. She was given 100 g of mannitol and a nicardipine drip was started with a systolic goal of <160 mmHg.

**Figure 2:**
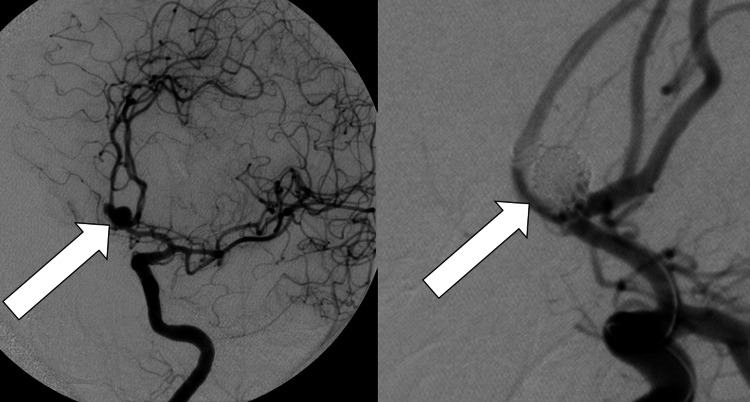
Angiogram of the ACoM artery aneurysm (left) and result after coiling (right). ACoM: anterior communicating.

On arrival, she was taken directly to the neuro-interventional radiology (NeuroIR) suite in preparation for the potential coiling of the aneurysm. Additionally, an emergent placement of an external ventricular drain (EVD) was performed that detected ICPs >20 mmHg. The aneurysm was subsequently successfully coiled (Figure 2). As the patient continued to have diminished clinical symptoms, the patient was admitted postoperatively to the neuro-intensive care unit for further monitoring. A targeted sodium level of 145-155 mEq/L was achieved along with saline infusion. This targeted level of sodium was performed due to the previous literature associating better outcomes in aSAH with the use of hypertonic saline [5]. Over the next few days, the ICP decreased to <20 mmHg and the patient’s pupils became symmetric, although the patient remained comatose.

**Figure 3:**
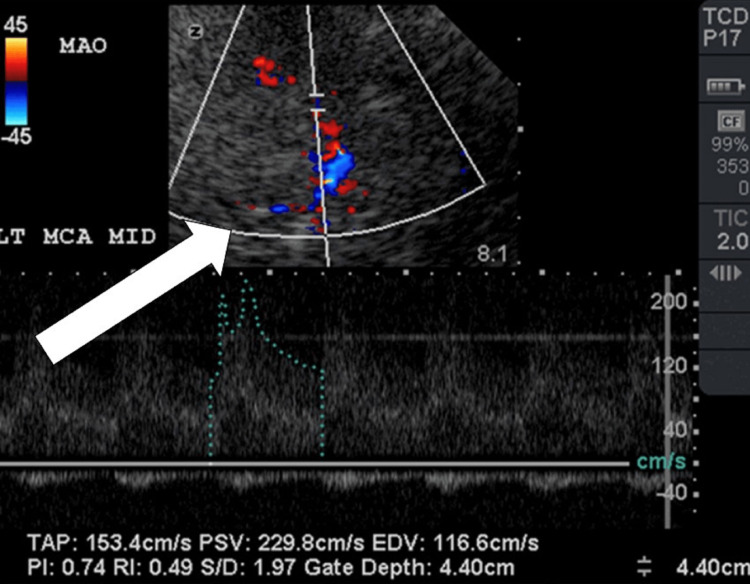
TCD on POD 7 showing moderate vasospasm of the MCA. MCA: middle cerebral artery; POD: postoperative day; TCD: transcranial doppler.

On a postoperative day (POD) 7, transcranial doppler (TCD) showed a mean flow velocity of 150cm/s of the left MCA, consistent with moderate vasospasm (Figure 3).

**Figure 4:**
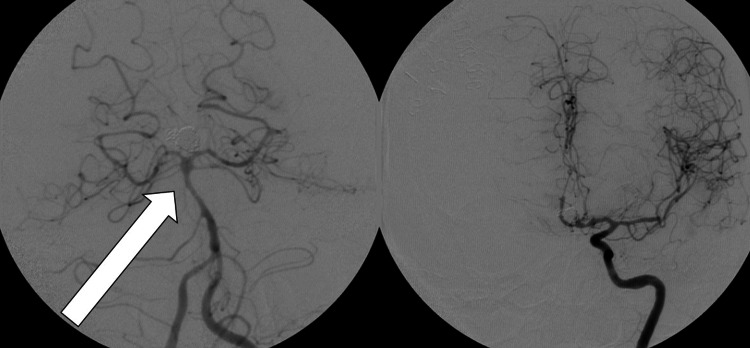
Severe basilar, ACA-A1 (left), and MCA-M1 (right) vasospasm. ACA: A1-anterior cerebral artery A1 segment; MCA: M1-middle cerebral artery M1 segment.

The patient was taken back to the NeuroIR suite, which revealed diffuse severe basilar and proximal circle of Willis vasospasm (ACA-A1, MCA-M1; Figure 4).

**Figure 5:**
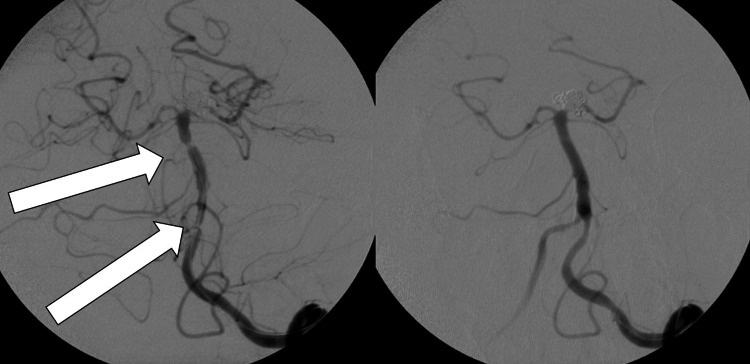
Balloon angioplasty performed for vasospasms for two consecutive days. Vasospasm of the basilar artery, A1 and A2 segment of the ACA, and left M1 segment of MCA (left). Post balloon angioplasty results of the basilar (right). ACA: anterior cerebral artery; MCA: middle cerebral artery.

Chemical spasmolytics were administered, and due to the severity of the vasospasm, balloon angioplasty of these arteries was successfully performed twice over two consecutive days (Figure 5). By POD 10, intermittent increases in ICP >40 mmHg became more frequent despite the use of mannitol and hypertonic saline, with imaging showing global cerebral edema (GCE). This is one disadvantage of having a high target for initial sodium levels, as in the setting of high ICPs, it is impossible to increase the sodium level even further with an initially high target range. Thus, the patient required low-dose sedation, neuromuscular paralysis, and targeted temperature management (TTM) with mild-moderate hypothermia (32-34 °C) to control her ICP below 20 mmHg.

On POD 24 (14 days total of TTM in hypothermia with repeated attempts to normalize the ICP), the ICP and temperature were sufficiently normalized. A few days later, the patient awoke from a coma and began to speak spontaneously. Her EVD was clamped and eventually removed. She rapidly weaned off the ventilator and was successfully transitioned to rehabilitation. At her three-month follow-up visit, she performed a Mini-Mental Status exam and a neurological exam with a translator, with a Modified Rankin Scale of 0.

## Discussion

Observations

The International Cooperative Study on the Timing of Aneurysm Surgery helped define mortality prognostication in aSAH patients including highlighting the role of early surgery timing in patient prognosis (<4 days versus 4-14 days) [1-3]. In the early phases of these studies, it was commonplace to hold off on surgical interventions on patients with poor grade aSAH until they showed neurological signs of recovery [3]. Currently, with the advancements in both endovascular and open-surgical techniques to treat aneurysms, the decision to operate depends on the clinical presentation of each patient [4]. In addition to aneurysm size and location, higher systolic blood pressure >160 mm Hg can also increase the risk of re-rupture of a previously secured aneurysm [6,7]. Lower systolic blood pressure thresholds between 140 and 160 mm Hg before intervention is critical to prevent rebleeding, with a higher threshold between 160 and 240 mm Hg after securing the aneurysm to prevent vasospasms [8,9].

Early management of high ICP and cerebral perfusion pressure (CPP) in poor-grade SAH is crucial towards achieving a favorable outcome. When CPP is absent (intracranial circulation arrest), GCE occurs, which leads to an impaired blood-brain barrier and disturbance of cerebral autoregulation [10,11]. When more volume is given or if blood flow is restored, more interstitial fluid leaks into the parenchyma (lower Hounsfield units due to increased brain water). GCE is an ominous sign that consequently requires CPP optimization, while simultaneously trying to keep ICP as low as possible [10,12]. The Lund therapy [13] also speaks to optimizing intracranial Starling’s laws with oncotic pressure and hydrostatic pressures.

CSF diversion techniques, such as an EVD placement, are commonly used in the neurocritical setting for ICP control. For refractory elevated ICPs that do not respond to CSF diversion techniques, one must perform a basic assessment of the head-neck position or tight bandages obstructing the outflow of the jugular veins [14]. Secondary ICP control can also be achieved with osmotherapy using mannitol or hypertonic saline [15,16]. When the ICP continues to be compromised with maximal medical therapy, emergent surgical craniectomy/decompression should be considered. Targeted temperature management for hypothermia reduces brain metabolism and thus reduces ICP. This has been shown to be effective in treating refractory ICPs in SAH and traumatic brain injury, although it has not been shown to definitively improve outcomes [9,17,18]. Overall, more studies are needed to fully establish an evidence-based guideline on the proper ICP/CPP management for poor-grade aSAH.

Lessons

Despite the lack of consensus for poor-grade aSAH, a multifaceted treatment plan can be deployed to reach favorable outcomes in patients. Regarding BP management, we propose keeping BP < 160 mmHg before intervention and subsequently being increased to 160-240 mmHg after an intervention to prevent the incidence of vasospasms. Additionally, due to the subtility of symptoms in patients with aSAH, deploying the use of TCD to detect vasospasms and subsequent treatment with balloon angioplasty was effective in the treatment of vasospasms. Lastly, postoperative treatment of fluctuating ICP levels was effectively managed with mannitol, hypertonic saline, TTM, and neuromuscular paralysis.

Ultimately, we would like to offer the following as major highlights of the present study: (i) despite clinical presentation with GCS of 4, we were able to see a favorable outcome with our patient presenting with poor grade aSAH; (ii) use of stringent multifaceted medical management, supported by literature, is imperative for dealing with patients with aSAH and profound neurological deficits; (iii) even with mannitol and saline use, ICP fluctuations and GCE were evident. However, with sedation and TTM, ICP was brought back to <20 mmHg.

## Conclusions

Despite the poor prognosis associated with clinical presentation, the authors show that with careful management of blood pressure and intracranial pressure, achieving a favorable outcome is possible. The use of a multimodal approach should be considered when managing poor-grade aSAH. Further research in aSAH and striving towards consensus in management would be of benefit to patients who present with poor prognoses. Consensus in management would help establish standardized treatment guidelines for this patient population.
